# Supplementary material for: VAPB ER-Aggregates, A Possible New Biomarker in ALS Pathology
Source: Cells. 2020 Jan 9;9(1):164. doi: 10.3390/cells9010164 (PMC7017080; doi:10.3390/cells9010164)
Supplement: Supplementary file 1 [file cells-09-00164-s001.pdf]

Supplementary Figure

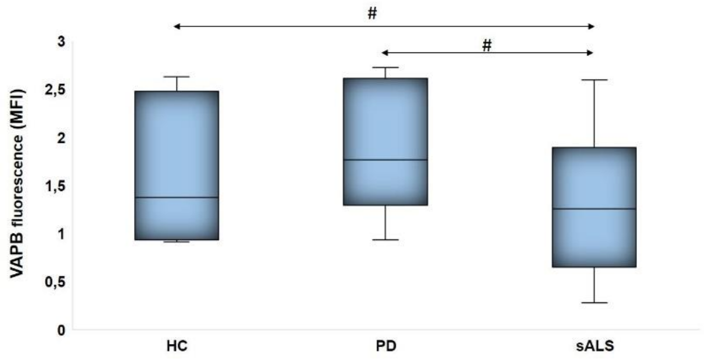

Figure S1. FCA assay performed with anti –human VAPB polyclonal antibody. The graph shows VAPB fluorescence signal not statistically significant in sALS patients respect to PD and HC. The data are expressed as medium intensity of fluorescence (MFI) in all patients and controls analyzed. *P<0.05, **P<0.005, # not significant.
